# Writing an effective literature review: Part II: Citation technique

**DOI:** 10.1007/s40037-018-0407-z

**Published:** 2018-03-02

**Authors:** Lorelei Lingard

**Affiliations:** Schulich School of Medicine & Dentistry, Health Sciences Addition, Western University, London, Ontario Canada

In the Writer’s Craft section we offer simple tips to improve your writing in one of three areas: Energy, Clarity and Persuasiveness. Each entry focuses on a key writing feature or strategy, illustrates how it commonly goes wrong, teaches the grammatical underpinnings necessary to understand it and offers suggestions to wield it effectively. We encourage readers to share comments on or suggestions for this section on Twitter, using the hashtag: #how’syourwriting?

This Writer’s Craft instalment is the second in a two-part series that offers strategies for effectively presenting the literature review section of a research manuscript. This piece argues that citation is not just a technical practice but also a rhetorical one, and offers writers an expanded vocabulary for using citation to maximal effect.

Many writers think of citation as the formal system we use to avoid plagiarism and acknowledge others’ work. But citation is a much more nuanced practice than this. Not only does citation allow us to represent the source of knowledge, but it also allows us to *position ourselves* in relation to that knowledge, and to *place that knowledge in relation to other knowledge*. In short, citation is how we artfully tell the story of what the field knows, how it came to that knowledge, and where we stand in relation to it as we write the literature review section to frame our own work. Seen this way, citation is a sophisticated task, requiring in-depth knowledge of the literature in a domain.

Citation is more than just referencing; it is how we represent the social construction of knowledge in a field. A citation strategy is any indication in the text about the source and nature of knowledge. Consider the following passage, in which all citation strategies are italicized:

Despite years of effort to teach and enforce positive professional norms and standards, *many reports of* challenges to medical professionalism *continue to appear, both in the medical and education literature and, often in reaction, in the lay press*.^1,2,3,4,5^ Examples of professional lapses dot the health care landscape: regulations are thwarted, records are falsified, patients are ignored, colleagues are berated.^2,4,6^ *The medical profession has articulated its sense* of what professionalism is *in a number of important position statements*.^7,8^ *These statements* tend to be *built upon* abstracted principles and values, such as the taxonomy *presented in the American Board of Internal Medicine’s (ABIM’s) Project Professionalism*: altruism, accountability, excellence, duty, honour, integrity, and respect for others.^7^

(From Ginsburg et al., The anatomy of a professional lapse [1])

In this passage, citation as referencing (in the form of Vancouver format superscript numbers) is used to acknowledge the source of knowledge. There are more than just references in this passage, however. Citation strategies also include statements that characterize the density of that knowledge (‘many reports’), its temporal patterns (‘continue to appear’), its diverse origins (‘both in the medical and education literature’), its social nature (‘often in reaction’), and its social import (‘important position statements’). Citation does more than just acknowledge the source of something you’ve read. It is how you represent the social nature of knowledge as coming from somewhere, being debated and developed, and having impact on the world [2]. If we remove all these citation strategies, the passage sounds at best like common sense or, at worst, like unsubstantiated personal opinion:

Despite years of effort to teach and enforce positive professional norms and standards, challenges to medical professionalism continue. Examples of professional lapses dot the health care landscape: regulations are thwarted, records are falsified, patients are ignored, colleagues are berated. Professionalism is a set of principles and values: altruism, accountability, excellence, duty, honour, integrity, and respect for others.

But perhaps you’ve been told that your literature review should be ‘objective’—that you should simply present what is known without taking a stance on it. This is largely untrue, for two reasons. The first involves the distinction between summary and critical summary. A summary is a neutral description of material, but a good literature review contains very little pure summary because, as we review, we must also judge the quality, source and reliability of the knowledge claims we are presenting [3]. To do this, we engage in *critical *summary, not only summarizing existing knowledge but offering a stance on it.

The second reason is that, even when we’re aiming for simple summary, a completely neutral presentation of knowledge claims is very difficult to achieve. We take a stance in ways we hardly even notice. Consider how the **verb** in each of these statements adds a flavour of stance to what is otherwise a summary of a knowledge claim in the field:

Anderson **describes** how the assessment is overly time-consuming for use in the Emergency Department.

Anderson **discovered** that the assessment was overly time-consuming for use in the Emergency Department.

Anderson **claims** that the assessment is overly time-consuming for use in the Emergency Department.

The first verb, ‘describes’, is neutral: it is not possible to ascertain the writer’s stance on the knowledge Anderson has contributed to the field. The second verb, ‘discovered’, expresses an affiliation or positive stance in the writer, while the third verb, ‘claims’, distances the writer from Anderson’s work. Even these brief summary sentences contain a flavour of critical summary. This is not a flaw; in fact, it is an important method of portraying existing knowledge as a conversation in which the writer is positioning herself and her work. But it should be done consciously and strategically. Tab. 1 offers examples to help writers think about how the verbs in their literature review position them in relation to existing knowledge in the field. Meaning is subject to context and these examples should only be taken as a guide: e. g., ‘suggests’ can be used to signal neutrality or distancing.

**Table 1:** Verbs to position the writer in relation to the literature being reviewed

Neutral about the knowledge		Affiliating with the knowledge		Distancing from the knowledge	
comments explains indicates notes describes	observes remarks states finds *suggests*	discovers reveals realizes understands	addresses argues recognizes identifies	assumes claims contends argues	hopes believes *suggests*

Most of us have favourite verbs that we default to almost unconsciously when we are writing—reports, argues, describes, studies, explains, asserts—but these verbs are not interchangeable. They each inscribe a slightly different stance towards the knowledge—not only the writer’s stance, but also the stance of the researcher who created the knowledge. It is critical to get the original stance right in your critical summary. Nothing irritates me more than seeing my stance mispresented in someone else’s literature review. For example, if I wrote a paragraph offering tentative reflections on a new idea, I don’t want to see that summarized in someone’s literature review as ‘Lingard argues’, when more accurate would be ‘Lingard suggests’ or ‘Lingard explored’.

Writers need to extend their library of citation verbs to allow themselves to accurately and persuasively position knowledge claims published by authors in their field. You can find many online resources to help extend your vocabulary: Tab. 2, adapted from one such online source [4], provides some suggestions. Tables like these should be thought of as tools, not rules—keep in mind that words have flexible meanings depending on context and purpose. This is why one word, such as **suggest** or **conclude**, can appear in more than one list.

**Table 2:** Verbs to represent the nature and strength of an author’s contributions to the literature

Verbs to report what an author DID	Verbs to report what an author SAID		Verbs to report an author’s OPINION	
analyse, assess, *conclude*, discover, describe, demonstrate, examine, explore, establish, find, identify, inquire, prove, observe, study, show	Weaker	Stronger	Weaker	Stronger
	comment, describe, note, remark, add, offer, *suggest*	affirm, emphasize, stress, maintain, stipulate, explain, *conclude*, identify, insist	accept, believe, consider, think, *suggest*, suspect, speculate	argue, assert, claim, contend, deny, recommend, reject, advocate, maintain

Knowledge is a social construction and it accumulates as researchers debate, extend and refine one another’s contributions. To avoid your literature review reading like a laundry list of disconnected ‘facts’, reporting verbs are an important resource. Tab. 3 offers a selection of verbs organized to reflect different relationships among authors in the field of knowledge being reviewed.

**Table 3:** Verbs to express relations among authors in the field

Depicting similar positions	Depicting contrasting positions	Depicting relating/responding positions
Taylor *acknowledges* Jackson’s claim that …	Taylor *refutes* Jackson’s claim that …	Taylor *adds* to Jackson’s claim that …
affirms, agrees, confirms, concurs, aligns, shares, echoes, supports, verifies, concedes, accepts	argues, disagrees, questions, dismisses, refuses, rejects, challenges contradicts, criticizes, opposes, counters, disputes	extends, elaborates, refines, builds on, reconsiders, draws upon, advances, repositions, addresses

Finally, although we have focused on citation verbs in this article, adverbs (e. g., similarly, consequently) and prepositional phrases (e. g., by contrast, in addition) are also important for expressing similar, contrasting or responding relations among knowledge claims and their authors in the field being reviewed.

In summary, an effective literature review not only summarizes existing knowledge, it also critically presents that knowledge to depict an evolving conversation and understanding in a particular domain of study. As writers we need to know when we are summarizing and when we are critically summarizing—summary alone makes for a literature review that reads like a laundry list of undigested ‘facts-in-the-world’. Finally, writers need to attend to the subtle power of citation verbs to position themselves and the authors they are citing in relation to the knowledge being reviewed. Broadening our catalogue of ‘go-to’ verbs is an important step in enlivening and strengthening our writing.
